# The Epidemiology of the Global Syndemic in Africa: New Evidence and Key Insights

**DOI:** 10.1007/s13679-026-00717-4

**Published:** 2026-04-27

**Authors:** Reuben Simfukwe, Felix P. Chilunga, Charles Agyemang

**Affiliations:** 1 Clinical Research Education and Management Services (CREAMS), Queen Elizabeth Central Hospital, Blantyre, Malawi; 2 Department of Public and Occupational Health, Amsterdam UMC, Amsterdam Public Health Research Institute, University of Amsterdam, Amsterdam, the Netherlands; 3 Department of Medicine, Johns Hopkins University School of Medicine, Baltimore, MD USA

**Keywords:** Global syndemic, Overnutrition, Undernutrition, Climate change, Africa

## Abstract

**Purpose of review:**

Over the past decade, Sub-Saharan Africa has entered a new phase in the evolution of the global syndemic of undernutrition, obesity, and climate change. Earlier reviews, largely based on evidence before 2018, documented the emergence of overlapping nutritional and environmental vulnerabilities. This review provides an updated synthesis of epidemiological evidence published between 2019 and 2025 to capture recent shifts in magnitude, distribution, and interactions of the syndemic components across Africa.

**Recent findings:**

Recent data indicate that adult obesity prevalence in the African region reached 12% in 2022, rising from 9% in 2010, with marked sex differences of approximately 17% among women and 7% among men. Overweight and obesity among children and adolescents remain lower but are increasing, with an estimated three million children affected in 2022. At the same time, undernutrition remains highly prevalent. Between 2019 and 2025, child stunting ranged from 9% to 46%, wasting from 4% to 43%, and anaemia from 19% to 61% across countries in the African region. Food insecurity increased from 17% in 2019 to 20% in 2025, affecting approximately 282 million people.

During the same period, climate-related shocks intensified. Extreme weather events affected 34 million people in Africa in 2023 alone, further disrupting food systems, reducing dietary diversity, and reinforcing reliance on energy-dense, nutrient-poor diets. Evidence now more clearly demonstrates that undernutrition, obesity, and climate stressors co-occur within the same populations and households, rather than representing sequential transitions.

**Summary:**

This update highlights advances in surveillance, analytical methods, and early warning systems since 2019, while identifying persistent gaps in longitudinal data, integration of nutrition and climate information, and policy implementation. Addressing the contemporary global syndemic in Africa requires context-specific, multisectoral strategies that prioritise double- and triple-duty actions to simultaneously reduce nutritional and climate vulnerability.

## Introduction

Over the past five decades, the nutrition landscape in Sub-Saharan Africa (SSA) has undergone a profound transformation. During the 1970 s and 1980 s, the region was predominantly characterised by severe undernutrition. Childhood stunting affected more than 40% of children in many countries [1], and micronutrient deficiencies were widespread [2]. By the late 1990 s and early 2000 s, rapid urbanisation, economic change, and dietary shifts led to a sharp rise in overweight and obesity, particularly among women living in urban areas, where prevalence in some countries reached as high as 40 to 50% [3, 4]. This transition marked the emergence of the double burden of malnutrition, in which undernutrition and overnutrition increasingly coexist within the same communities, households, and even individuals [5].

In the 2010 s and 2020 s, an additional and increasingly prominent source of vulnerability emerged. Climate change, manifested through recurrent droughts, floods, and extreme heat, has disrupted food systems and contributed to persistent cycles of food insecurity [6, 7]. Currently, approximately 282 million people in Africa, nearly one in five, experience hunger, while dietary patterns have shifted toward greater consumption of cheap, energy-dense, and highly processed foods [8]. These dynamics are interlinked. Climate shocks reduce access to diverse and nutritious foods, intensifying undernutrition and increasing reliance on nutrient-poor diets, while rising obesity continues alongside rapid urbanisation and lifestyle change [9].

Together, these developments reflect what The Lancet Commission in 2019 conceptualised as the global syndemic, defined by the synergistic and mutually reinforcing interaction of undernutrition, obesity, and climate change [10]. Prior to this formal definition, several African-focused reviews and empirical studies published between 2010 and 2018 had already documented how these domains were unfolding concurrently. For example, a systematic review by Steyn and Mchiza (2014) reported persistently high levels of stunting, often ranging from 17% to 40%, alongside rising overweight and obesity, particularly among women in urban areas of South Africa, Ghana, and Kenya [4]. Similarly, Popkin, Adair, and Ng (2012) described a rapid nutrition transition in many African countries, characterised by increased consumption of refined grains, vegetable oils, and sugar-sweetened beverages [3]. Empirical evidence from Nairobi and Dar es Salaam further demonstrated that urban poor populations were simultaneously experiencing chronic undernutrition and rising obesity [11].

Climate-focused literature from the same period also revealed early syndemic patterns. Grace et al. (2012) showed that drought-related crop failures in Ethiopia were associated with reduced dietary diversity and significant increases in child undernutrition [12]. Haggblade et al. (2017) documented how climate variability in the Sahel undermined agricultural productivity and pushed households toward cheaper staple-based diets with low micronutrient content [13]. Studies by Lobell et al. (2011) and Niang et al. (2014) further demonstrated that projected temperature increases and rainfall instability were already translating into measurable reductions in yields of key African crops as maize, sorghum, and millet, intensifying food insecurity [6, 14].

Collectively, these earlier reviews and empirical studies recognised that undernutrition, obesity, and climate pressures were beginning to intersect within the same populations and several adopted syndemic or systems-oriented perspectives. However, they relied on evidence available up to the late 2010 s and therefore predated many of the major climatic, dietary, and demographic shifts now reshaping the region. They did not capture the increasing frequency of climate shocks over the past decade, the accelerating penetration of ultra-processed foods into African food environments, or the most recent Demographic and Health Survey cycles, Food and Agriculture Organization food security updates, and Intergovernmental Panel on Climate Change assessments.

Given the pace and complexity of these changes in the past half decade, an updated synthesis is urgently required to reflect the contemporary dynamics of the global syndemic in SSA. This narrative review therefore provides a comprehensive and timely update, integrating the most recent evidence on undernutrition, overweight and obesity, and climate pressures to inform research, policy, and multisectoral action in the region.

## Methodology

This narrative review synthesised evidence on the intersecting burdens of undernutrition, overweight and obesity, and climate change in SSA within the framework of the global syndemic. The review focused primarily on literature published over the past half-decade (2019–2025), while selectively drawing on earlier seminal studies to contextualise historical trends.

A structured search was conducted in PubMed, Web of Science, and Scopus using keywords related to nutrition (e.g., undernutrition, stunting, wasting, micronutrient deficiency, overweight, obesity), climate change (e.g., climate variability, drought, floods, extreme heat), food systems, and SSA. Grey literature was identified through targeted searches of reports from the World Health Organization (WHO), Food and Agriculture Organization, UNICEF, the World Bank, the Intergovernmental Panel on Climate Change, and Demographic and Health Survey programmes.

Included sources comprised systematic reviews, observational and modelling studies, national and regional surveillance reports, and policy documents providing population-level evidence on nutritional outcomes, food insecurity, or climate-related exposures in African settings. Evidence was synthesised narratively and organised into thematic domains aligned with the manuscript structure, including nutrition trends, climate stressors, food systems interactions, and health and equity consequences.

This approach aimed to integrate diverse and rapidly evolving evidence, identify converging patterns and reinforcing pathways, and provide an updated synthesis of the global syndemic in SSA.

## Recent Trends in the Global Syndemic in Africa (2019 to 2025)

### Overnutrition

According to the WHO, overweight and obesity are defined as abnormal or excessive fat accumulation that presents a risk to health. In adults, a body mass index (BMI) of 25 kg/m² or higher indicates overweight, while a BMI of 30 kg/m² or higher indicates obesity. Among children and adolescents, age- and sex-specific body mass index thresholds are used. Overweight and obesity arise from complex interactions between obesogenic environments, behavioural and psychosocial factors, and genetic susceptibility. These conditions substantially increase the risk of non-communicable diseases (NCDs), including type 2 diabetes and cardiovascular disease, and contribute to premature mortality. In children, overweight and obesity are also associated with psychosocial consequences that negatively affect quality of life and educational performance [15]. The economic burden of overweight and obesity is considerable and is projected to increase substantially in the coming decades [16].

Between 2019 and 2025, the prevalence of overweight and obesity in Africa has continued to rise, posing a growing public health challenge. WHO estimates indicate that adult obesity prevalence in the African Region reached 12% in 2022, increasing from 9% in 2010 [15]. Obesity prevalence was higher among women, at approximately 17%, compared with about 7% among men in 2022 [15]. However, substantial variation exists across countries. Among adult women, the prevalence of overweight and obesity was lower in Ethiopia at around 20% and markedly higher in Cape Verde at approximately 57% [17–20]. Among adult men, combined overweight and obesity prevalence ranged from about 21% in Ghana to 41% in Cape Verde, although several studies did not report separate estimates for overweight and obesity [19, 21].

Urban residence is consistently associated with higher levels of overnutrition. In Cape Verde, 51% of adults living in urban areas were overweight or obese by 2020, compared with 47% in rural areas [19]. Similar patterns have been observed in Ghana, where 27% of men in urban areas were overweight or obese compared with 12% in rural areas, and among women, where prevalence reached 44% in urban settings compared with 26% in rural settings during the period 2019 to 2023 [17, 21]. These disparities reflect differential exposure to energy-dense diets, sedentary lifestyles, and urban food environments.

Among children and adolescents younger than 19 years, the prevalence of overweight and obesity remains lower than in adults but is increasing. Studies published between 2019 and 2025 report prevalence estimates ranging from 3% in Ethiopia to 18% in Zambia. Across these studies, overweight prevalence ranged from 9% to 13%, while obesity ranged from 5% to 6% [22–27]. WHO estimates indicate that approximately three million children in Africa were overweight or obese in 2022, representing an increase of nearly 12% since 2000 [15]. This upward trend suggests a growing future burden of adult obesity and related NCDs.

Projections from the World Obesity Federation World Obesity Atlas 2022 indicate that, without effective intervention, the prevalence of overweight and obesity among African adults is expected to increase sharply by 2030. These projections underscore the urgency of implementing preventive strategies to avert further escalation of obesity-related morbidity, mortality, and health system strain across the continent [28].

### Undernutrition and Food Insecurity

Undernutrition comprises four main forms: wasting, defined as low weight for height; stunting, defined as low height for age; underweight, defined as low weight for age; and micronutrient deficiencies, reflecting inadequate intake of essential vitamins and minerals. Wasting substantially increases the risk of mortality through heightened susceptibility to infections, while stunting impairs physical growth and cognitive development in children. Micronutrient deficiencies further compromise growth, development, and physiological function by limiting the body’s capacity to produce enzymes, hormones, and other essential compounds [29]. Undernutrition most commonly arises in the context of food insecurity, defined as a lack of regular access to sufficient, safe, and nutritious food for normal growth, development, and an active and healthy life. Food insecurity is shaped by food availability, access, utilisation, and stability [30].

According to estimates from the WHO and UNICEF, undernutrition in Africa remains high relative to global averages. Although prevalence declined between 2012 and 2024, recent data suggest that these gains are stalling or reversing in several settings [31]. Across studies published between 2019 and 2025 that focused on children and adolescents younger than 19 years, the overall prevalence of undernutrition ranged widely, from 10.4% to 58.4%. Wasting prevalence reported in ten studies ranged from 3.8% in Tanzania to 42.9% in Ethiopia [20, 27, 32–39]. Stunting prevalence reported in thirteen studies ranged from 9.1% in South Africa to 45.8% in Ethiopia [20, 26, 27, 32, 34–36, 39–44]. Underweight prevalence reported in six studies ranged from 12% in Ghana to 23.3% in Ethiopia [20, 26, 34–36, 44]. Anaemia prevalence reported in five studies ranged from 19.2% in South Africa to 60.5% in Tanzania [26, 32, 43, 45, 46]. These estimates are broadly consistent with findings from studies published before 2019, indicating limited overall progress in reducing undernutrition over time [47–50]. Notably, none of the reviewed studies examined adult undernutrition, highlighting an important gap in current evidence.

Micronutrient deficiencies remain widespread across the region. More than half of all children and approximately two-thirds of non-pregnant women of reproductive age in Eastern and Southern Africa are deficient in one or more essential vitamins and minerals, reflecting persistently low dietary diversity [51–53]. However, population-level data on micronutrient status remain sparse. Among studies published between 2019 and 2025, one South African study reported vitamin D deficiency in 28.5% of adults, while a study from Uganda reported vitamin A deficiency in 8.9% of children younger than five years [36, 54]. These findings indicate substantial micronutrient gaps that remain poorly characterised at the population level.

Food insecurity has increased across Africa in recent years. The 2025 State of Food Security and Nutrition in the World report documented a rise in food insecurity prevalence from 17% in 2019 to 19% in 2022, reaching approximately 20% in 2025. If current trends persist, Africa is projected to account for around 60% of the global food-insecure population and nearly half of all undernourished people by 2030 [55–57]. Considerable heterogeneity exists across countries. Between 2019 and 2025, food insecurity prevalence ranged from 17% in Burkina Faso to as high as 88% in Sierra Leone, reflecting differences in food systems, economic conditions, governance, and climate vulnerability [58–65].

Armed conflict further exacerbates food insecurity and undernutrition in many parts of Africa. Evidence indicates that prolonged conflict substantially increases the likelihood of child undernutrition, with odds rising up to threefold in some settings, particularly in Central Africa [66]. As a result, undernutrition increasingly coexists with rising overweight and obesity. Between 2019 and 2025, studies reported a community-level double burden prevalence of approximately 12% in Tanzania and household-level prevalence ranging from 9% in Senegal to 17% in Ethiopia [20, 26, 67]. These patterns highlight a rapidly evolving nutrition landscape in which persistent undernutrition overlaps with increasing overweight and obesity across populations.

### Climate Change and Environmental Stressors

Climate change refers to long-term shifts in temperature and weather patterns, primarily driven by increased concentrations of greenhouse gases such as carbon dioxide and methane, largely originating from agriculture, energy production, and industrial activities [68]. In addition to greenhouse gases, particulate matter generated from biomass burning, agricultural practices, and household energy use acts as a short-lived climate forcer that accelerates climate change and directly affects human health [7]. Together, these environmental changes disrupt ecosystems that underpin clean air, safe drinking water, nutritious food supplies, and adequate shelter, posing growing risks to global health. Under current trajectories, climate change is projected to cause approximately 250,000 additional deaths annually between 2030 and 2050 from malnutrition, malaria, diarrhoeal disease, and heat stress alone [69].

According to assessments by the Intergovernmental Panel on Climate Change, Africa contributes only 2 to 3% of global greenhouse gas emissions but is already experiencing disproportionately severe impacts of climate change [7]. These impacts are amplified by low adaptive capacity in many African countries, including limited climate-resilient infrastructure, inadequate early warning systems, constrained financing for adaptation, and heavy reliance on rain-fed agriculture. As a result, climate risks increasingly threaten livelihoods, food security, health systems, and long-term economic development across the continent.

The scale of climate-related impacts has intensified in recent years. Between 2022 and 2023, the number of people affected by extreme weather events in Africa increased from 19 million to 34 million [70]. In 2023 alone, floods affected 23 countries, cyclones struck three countries, and five countries experienced heatwaves with temperatures exceeding seasonal averages by more than 20 °C [70]. Extreme heat in parts of northern Africa triggered wildfires, while severe drought and famine affected countries including Djibouti, Ethiopia, Kenya, Mauritania, Niger, and Somalia. These crises left approximately 23 million people food insecure and contributed to acute malnutrition among an estimated four million children [70, 71]. The true scale of these impacts is likely underestimated, as Africa has the lowest density of meteorological monitoring stations globally [70].

Urbanisation further interacts with climate and nutritional risks. Over the past two decades, Africa has experienced rapid urban growth, with an average annual increase of approximately 3.5% [72]. The proportion of the population living in urban areas rose from 36% in 2010 to 45% in 2024 and is projected to reach 50% by 2030 and 60% by 2050 [72]. Rapid and often unplanned urban expansion has strained infrastructure and services, widened social inequalities, and increased environmental pressures [72]. Concurrently, a nutrition transition has occurred, characterised by shifts away from traditional diets toward energy-dense, processed, and animal-source foods [73]. This transition has exacerbated the double burden of malnutrition, with undernutrition and overweight and obesity increasingly coexisting in both urban and rural settings [73].

### Interactions Between Components Forming Global Syndemic in Africa

**Fig. 1:**
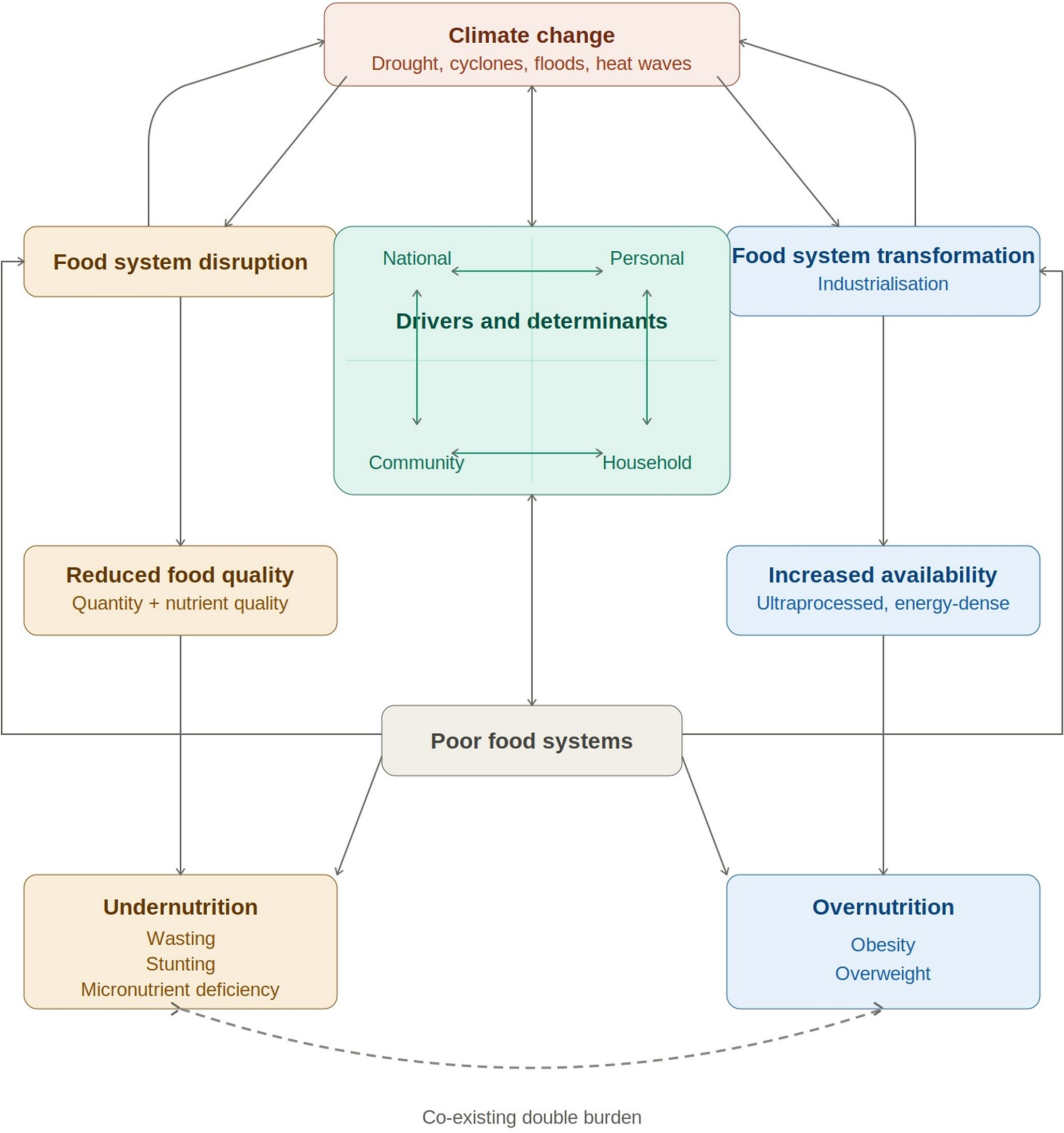
Interactions between components forming global syndemic in Africa. Climate change interacts bidirectionally with national, community, household, and personal drivers and determinants, and drives two parallel pathways leading to the double burden of malnutrition. On the left, food system disruption reduces food quality, quantity, and nutrient quality, resulting in undernutrition (wasting, stunting, and micronutrient deficiency). On the right, food system transformation through industrialisation increases the availability of ultraprocessed and energy-dense foods, resulting in overnutrition (obesity and overweight). Poor food systems mediate and reinforce both pathways through bidirectional interactions with the drivers and determinants, and feed back into food system disruption and transformation. The dashed arc indicates the co-existence and mutual reinforcement of undernutrition and overnutrition as a double burden

The components of the global syndemic interact through multiple, reinforcing pathways, most clearly understood through a food systems lens (Fig. 1). The Lancet Commission identified unhealthy and unsustainable food systems as a central driver linking undernutrition, overweight and obesity, and climate change [10]. In this framework, climate change disrupts food production and distribution through extreme weather events, reducing both the quantity and nutritional quality of foods, particularly in low-income and agrarian settings. These disruptions disproportionately affect food-insecure populations, increasing the risk of undernutrition [10].

At the same time, structural changes in food systems have favoured the production, processing, and distribution of energy-dense, nutrient-poor foods. Advances in agricultural productivity and food processing have prioritised refined grains, vegetable oils, and ultra-processed foods over diverse and nutrient-rich crops [10, 74]. This shift has increased the availability and affordability of highly processed diets, contributing to micronutrient deficiencies and rising overweight and obesity [75]. These same food system changes, together with expanding transportation networks and urban land use, contribute to greenhouse gas emissions, reinforcing climate change and creating a self-perpetuating cycle [10].

Africa is particularly vulnerable to these interacting pathways. Although the continent contributes only a small fraction of global greenhouse gas emissions, the predominance of rain-fed agriculture makes food production highly sensitive to climatic variability [7]. Extreme weather events have been linked to persistently high levels of child undernutrition, largely through their effects on food availability, access, and prices [76]. Concurrently, rapid and often unplanned urbanisation has altered diets and physical activity patterns, increasing exposure to obesogenic food environments while socioeconomic inequalities sustain pockets of undernutrition within the same urban settings [77].

Empirical studies provide insight into how climate variability affects nutritional outcomes. In Malawi, rainfall was positively associated with child overweight and reduced stunting, while rising temperatures were associated with adverse nutritional trends among children younger than five years [78]. In Burkina Faso, increased rainfall variability combined with declining average rainfall was associated with higher rates of child stunting after accounting for socioeconomic factors [79, 80]. In contrast, improved crop yields in Uganda have been associated with gains in child height for age, illustrating the context-specific nature of climate–nutrition interactions [81].

Recent country-level evidence highlights the cumulative effects of these intersecting pressures. In Ethiopia, household-level double burden of malnutrition has increased in recent years alongside recurrent climate shocks. Although child wasting declined from approximately 16% in 2000 to 9.1% in 2019, overweight and obesity increased from less than 1% to at least 3% over the same period [25, 82]. These nutritional shifts coincided with dietary transitions toward more processed foods and with prolonged drought linked to consecutive failed rainy seasons, resulting in food insecurity, population displacement, and adverse health outcomes [74, 83]. Similar dynamics are observed in Somalia, where food systems are shaped by the combined effects of climate extremes, conflict, displacement, and weak governance rather than isolated shocks [39].

Together, these examples illustrate how climate change, food systems, and social and economic inequalities interact to produce overlapping burdens of undernutrition and overweight and obesity. As climate risks intensify, these interactions are likely to further entrench nutritional inequities and widen health disparities across SSA.

### Drivers and Determinants of Global Syndemic in Africa

**Fig. 2:**
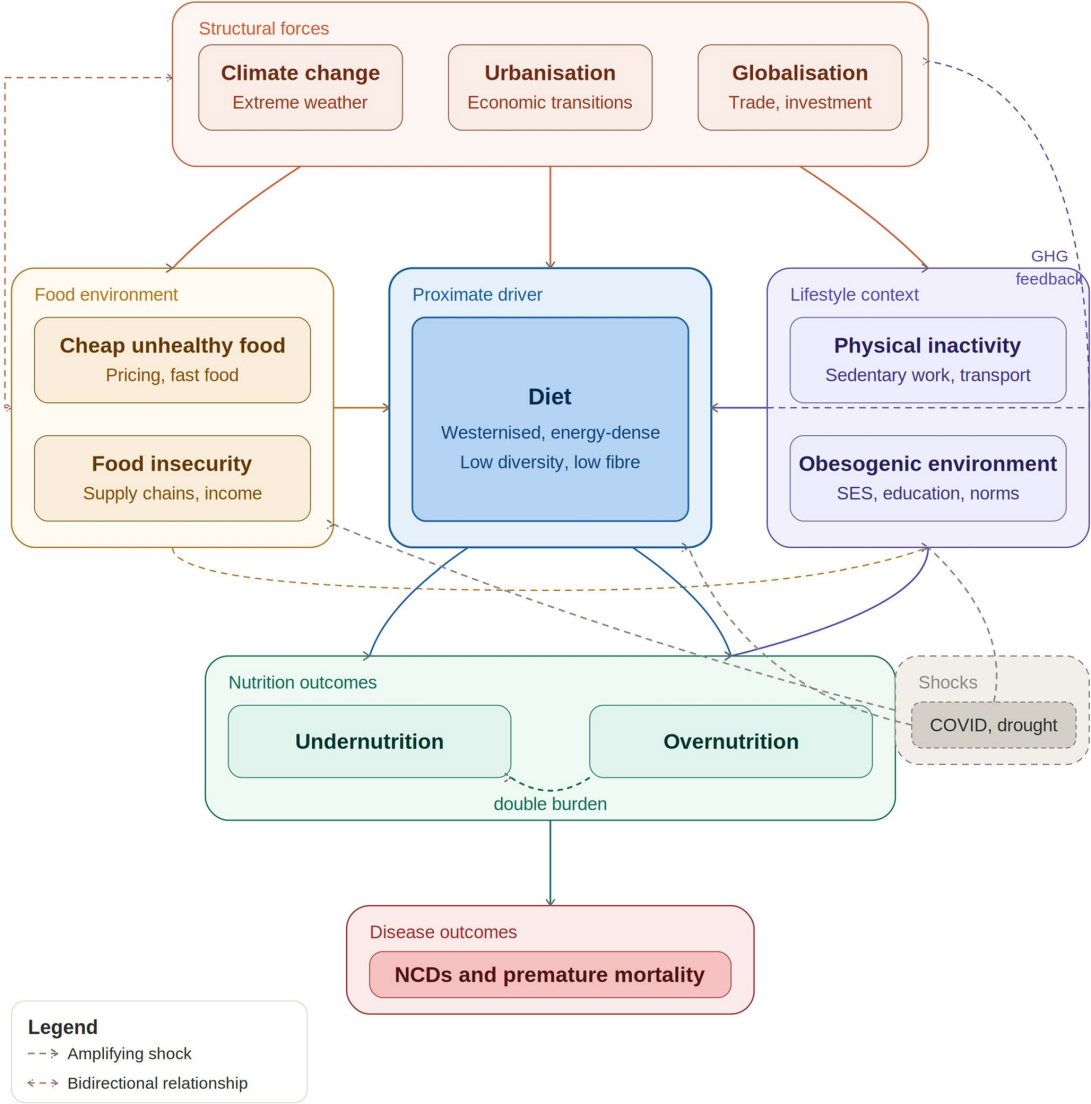
Drivers and determinants of global syndemic in Africa. The global syndemic in Africa arises from interacting determinants across structural, food system, and individual levels, with diet as the central proximate driver. Structural forces including climate change, urbanisation, economic transitions, and globalisation shape both the food environment and lifestyle context. Climate change and food systems interact bidirectionally, with extreme weather disrupting food availability while lifestyle changes reinforce climate pressures through greenhouse gas (GHG) emissions. Cross-cutting shocks including infectious disease outbreaks and drought amplify vulnerabilities across multiple levels. These dynamics produce a co-existing double burden of undernutrition and overnutrition, converging on non-communicable diseases and premature mortality. Dashed arrows indicate amplifying or feedback relationships; double-headed arrows indicate bidirectional relationships. GHG: greenhouse gas emissions

The global syndemic in Africa is shaped by interacting determinants operating at individual, household, community, and structural levels, with diet representing a central and proximate driver. These determinants form a system with reinforcing feedback loops, linking social, economic, and environmental factors to nutritional outcomes (Fig. 2).

At the individual level, dietary choices are influenced by nutrition knowledge, educational attainment, income, place of residence, and lifestyle. Evidence from SSA consistently shows higher prevalence of overweight and obesity among individuals living in urban areas, a pattern closely linked to shifts from traditional diets toward westernised dietary patterns characterised by high intake of processed foods, saturated fats, refined sugars, and low fibre [10, 17, 24]. Urbanisation has also reduced opportunities for physical activity through increased reliance on motorised transport and sedentary occupations, further contributing to positive energy balance These lifestyle changes simultaneously contribute to greenhouse gas emissions, reinforcing climate pressures. Higher educational attainment has been associated with increased risk of overweight and obesity in several African settings, reflecting improved socioeconomic status, urban residence, and greater exposure to obesogenic environments [10, 17, 24].

Evidence on household, community, and national-level determinants remains limited, as most studies focus on individual-level factors. Available studies indicate that food affordability, availability, and pricing strongly influence dietary patterns. In South Africa, the cost of highly processed and unhealthy foods has been reported to be up to 70% lower than that of healthier alternatives, contributing to reduced dietary diversity and increased consumption of nutrient-poor foods [75]. This price differential is compounded by the rapid expansion of fast-food outlets, which outnumber healthier food outlets in many urban areas [84, 85]. Similar associations have been observed in Tanzania, where increased availability of unhealthy foods was associated with higher odds of overweight and obesity [18].

Cross-cutting shocks further amplify these determinants. Infectious disease outbreaks represent one such shock, although empirical evidence in SSA remains largely limited to the COVID-19 pandemic, with only limited comparisons to earlier Ebola outbreaks [60–63, 86, 87]. The COVID-19 pandemic disrupted food supply chains, household incomes, and opportunities for physical activity, with documented increases in food insecurity across multiple African countries [60–63, 86, 87]. The impact of the COVID-19 pandemic on food security has been reported to be more severe than that observed during the 2014 Ebola outbreak, largely due to differences in the speed of transmission and the scale and nature of response measures implemented [88]. In Ethiopia, the combined effects of the pandemic, drought, and population displacement further worsened nutritional vulnerability in affected regions [60]. Evidence linking cholera outbreaks to food security remains limited. While a study by Elnaiem et al. (2023), reported an association between food insecurity and increased cholera risk [89], evidence on changes in overweight and obesity during this period remains limited, highlighting a gap in current research.

At the regional and structural level, climate change exerts a bidirectional influence on food systems and nutritional outcomes. Extreme weather events disrupt agricultural production, food availability, and prices, reinforcing cycles of food insecurity and reliance on inexpensive, energy-dense foods. These dynamics interact with urbanisation and economic transitions, producing a system in which nutritional outcomes are shaped not by single exposures but by interconnected forces spanning local to regional scales.

Taken together, these nested determinants illustrate that the global syndemic in Africa arise from complex systems rather than isolated behaviours. Addressing these drivers therefore requires coordinated, multisectoral interventions that target structural conditions alongside individual-level factors.

### Health and Equity Consequences of the Global Syndemic in Africa

**Fig. 3:**
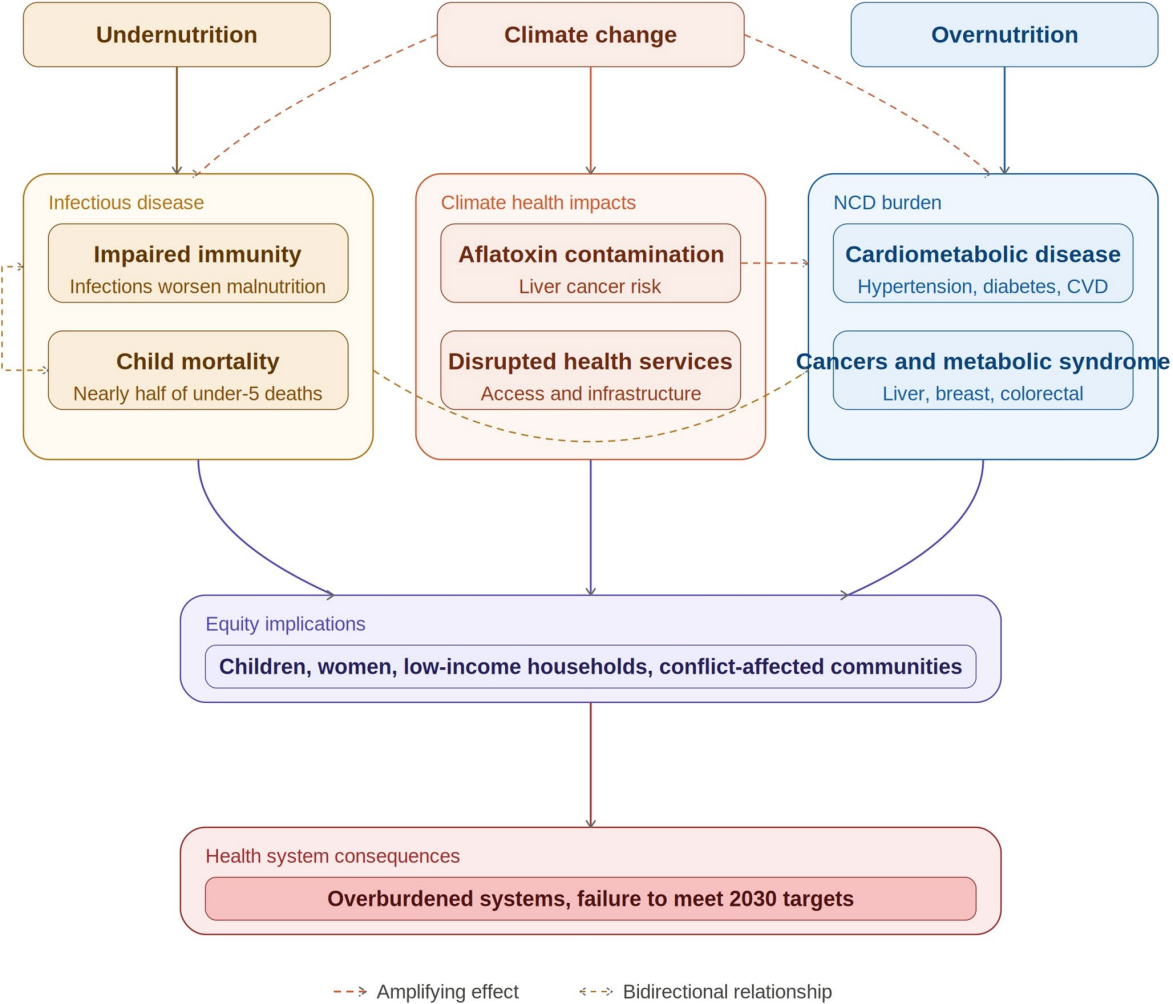
Impact of global syndemic on health and wellbeing. Undernutrition, climate change, and overnutrition produce overlapping health burdens. Undernutrition impairs immunity and worsens infectious disease susceptibility, accounting for nearly half of under-five deaths, and increases cardiometabolic disease risk in adulthood. Overnutrition drives hypertension, diabetes, cardiovascular disease, and obesity-related cancers. Climate change amplifies both pathways through aflatoxin contamination and disruption of health services. These burdens fall disproportionately on vulnerable populations, overburdening health systems and threatening Africa’s ability to meet 2030 health and nutrition targets

The combined effects of obesity, undernutrition, and climate change have wide-ranging and increasingly visible consequences for health and equity in Africa (Fig. 3). These interacting burdens place growing pressure on health systems that are already constrained and disproportionately affect populations facing social and economic disadvantage. Undernutrition increases susceptibility to infectious diseases and delays recovery, while overweight and obesity drive the rising burden of NCDs. Climate change further exacerbates these risks through direct and indirect pathways affecting nutrition, disease exposure, and access to health services.

In 2021, an estimated 43 million deaths worldwide were attributed to NCDs, with nearly 73% occurring in low- and middle-income countries [90]. Overweight and obesity accounted for more than 3.5 million of these deaths, reflecting their central role as risk factors for cardiovascular disease, type 2 diabetes, and other chronic conditions [91]. Evidence from Africa mirrors these global trends. A study conducted in The Gambia in 2019 showed that overweight and obesity significantly increased the odds of hypertension and diabetes, findings consistent with earlier studies from other African countries [92–95]. Our own Pros-RODAM studies in 2022 in Ghana also showed that hypertension had reached 37% in rural Ghana, 54% in urban Ghana of which obesity was a significant contributor [96]. Rising obesity has also been linked to increased risk of several cancers, including liver, oesophageal, breast, prostate, colorectal, ovarian, uterine, pancreatic, kidney, gallbladder, and thyroid cancers, with growing evidence of increasing incidence in African settings [97]. Similarly, climatic conditions characterised by high temperatures and humidity have been associated with increased aflatoxin contamination of stored foods, contributing to elevated liver cancer risk in some African regions [98].

At the opposite end of the malnutrition spectrum, undernutrition remains a major driver of child morbidity and mortality. According to the Barker hypothesis, adverse nutrition in early life, including prenatally, as measured by birth weight, increases susceptibility to the metabolic syndrome a cluster of conditions comprising of obesity, diabetes, insulin insensitivity, hypertension, and hyperlipidemia, leading to complications that include coronary heart disease and stroke [99]. Evidence supporting this hypothesis in lower income countries including African countries is unfolding. Both food insecurity, especially exposure to famine during childhood and childhood malnutrition have shown to be an increased risk of developing cardiometabolic NCDs including; obesity, diabetes, hyperlipidemia, hypertension and other cardiovascular diseases [100–103]. However, evidence on the biological mechanisms remain limited and incompletely understood in Africa settings.

Undernutrition has also shown a complex bidirectional relationship with infectious diseases. Undernutrition affects immunity and infections contribute to malnutrition [104]. For example, in SSA, children with diarrhoeal diseases have been shown to have increased odds of developing undernutrition [105]. Similarly, in Zimbabwe, approximately 28% of households with at least one HIV-positive member had a stunted child compared with 24% of households without HIV- positive member [106]. Conversely, micronutrients deficiency, particularly, vitamin E and iron, ware associated with increase odds of malaria infection in Rwanda [107]. Likewise, study done in South Africa found that undernourished children exhibited lower vaccine-induced antibody responses, increasing their vulnerability to infections such as measles [108].

Nearly half of all deaths among children under 5 years of age are linked to undernutrition [109], with most of these deaths resulting from infectious diseases. In multinational study done in Ethiopia, Kenya, Mali, Mozambique, Sierra Leone and South Africa, 89% of deaths among undernutrition children were attributed to infectious cause. Moreover, the odds of mortality in malnourished children was twice as high as those of children who died from other causes [110, 111].

These health impacts are unevenly distributed, reinforcing existing social and health inequities. Vulnerable populations, including children, women, low-income households, and communities affected by conflict or displacement, bear a disproportionate share of the syndemic burden [66, 112–114]. Under current trajectories, Africa is unlikely to meet the nutrition and health targets set by the WHO, the United Nations, and the African Union for 2030 [115–117]. Addressing the health and equity consequences of the global syndemic therefore requires integrated strategies that simultaneously reduce nutritional vulnerability, mitigate climate risks, and strengthen health system resilience.

### Technological and Methodological Advances in Addressing the Global Syndemic in Africa

Recent years have seen important advances at the global level in monitoring and addressing the obesity syndemic and its interaction with climate change. These advances include strengthened nutritional surveillance systems, application of artificial intelligence for risk prediction, and expanded use of satellite-based climate and environmental monitoring. In the United States, the Centers for Disease Control and Prevention operate repeated population-based surveys across multiple surveillance platforms to track long-term trends in obesity, diet, and physical activity, enabling evaluation of policies and interventions over time [118]. These systems are complemented by advanced meteorological monitoring and early warning mechanisms that support preparedness for extreme weather events and climate-related health risks [70]. Globally, satellite platforms operated by the European Space Agency and the National Aeronautics and Space Administration provide high-resolution data on particulate matter, land use, vegetation, and atmospheric conditions, supporting integrated assessment of air quality, climate dynamics, and population health impacts [119, 120]. In parallel, international agencies increasingly apply integrated food system and health impact models to assess how dietary patterns influence obesity and NCDs, informing global dietary guidelines and climate mitigation strategies [118].

In Africa, advances are emerging but remain more limited in scale and integration. District Health Information Software 2 is widely used to collect, manage, analyse, and visualise routine health data, although nutrition- and climate-specific indicators are often insufficiently detailed [121]. The WHO STEPwise approach to Surveillance provides periodic data on NCDs risk factors, including obesity, across several African countries [122]. African researchers have also developed innovative tools such as the Nutrition Early Warning Systems, which integrates satellite imagery, rainfall, temperature, and vegetation indices to anticipate crop nutritional value and identify populations at risk of malnutrition under changing climate conditions [123, 124].

Analytical innovations are increasingly being applied to understand spatial and temporal patterns of malnutrition and climate vulnerability. Geographic Information Systems have revealed clustering of undernutrition and climate risk, identifying hotspots such as child wasting in Ethiopia’s Somali region and northeastern Mozambique [33, 40]. Machine learning approaches, including artificial neural networks, have been used to predict stunting with moderate accuracy in settings such as Rwanda, demonstrating the potential of data-driven tools to support targeted interventions and early action [125].

Despite these advances, major gaps persist. Surveillance systems in Africa often lack longitudinal coverage, fine geographic resolution, and integration across nutrition, health, and climate domains. Many digital and predictive tools remain at pilot stage, with limited evaluation of effectiveness, scalability, or policy uptake. Compared with high-income settings, there remains weak linkage between data generation and decision-making, as well as insufficient investment in early warning systems and climate-resilient food system monitoring. Strengthening these capacities is essential to translate emerging technological advances into timely, context-specific responses to the obesity syndemic and the broader global syndemic in Africa.

### Policy Responses and Future Directions

Building on recent technological and methodological advances, policy responses to the intersecting burdens of undernutrition, obesity, and climate change have expanded at both global and national levels. Most countries have adopted national nutrition strategies, NCD action plans, and food security or agricultural policies, often aligned with global frameworks such as the WHO Global Action Plan on NCDs and the UN Decade of Action on Nutrition [126, 127]. In Africa, these policies have historically prioritised undernutrition through maternal and child nutrition programmes, school feeding schemes, and nutrition-sensitive social protection [128].

As overweight and obesity have increased, some African countries have introduced measures targeting unhealthy food environments. These include sugar-sweetened beverage taxes, restrictions on marketing unhealthy foods to children, and efforts to improve dietary quality within school feeding programmes [129]. While early evidence suggests potential benefits, implementation remains uneven, enforcement capacity is limited, and formal evaluation of health and equity impacts is scarce. Importantly, most national strategies continue to address undernutrition, obesity, and climate change as separate challenges, despite their growing convergence [129].

Evidence from high-income settings indicates that integrated food system policies can deliver co-benefits for health and the environment. Modelling studies suggest that food-based dietary guidelines aligned with both health and sustainability objectives could reduce premature mortality from NCDs by approximately 15% while lowering greenhouse gas emissions by around 13% [130]. In African contexts, however, projected gains are smaller due to high baseline mortality, persistent food insecurity, and structural constraints within food systems, underscoring the need for contextual adaptation rather than direct policy transfer [129].

**Fig. 4:**
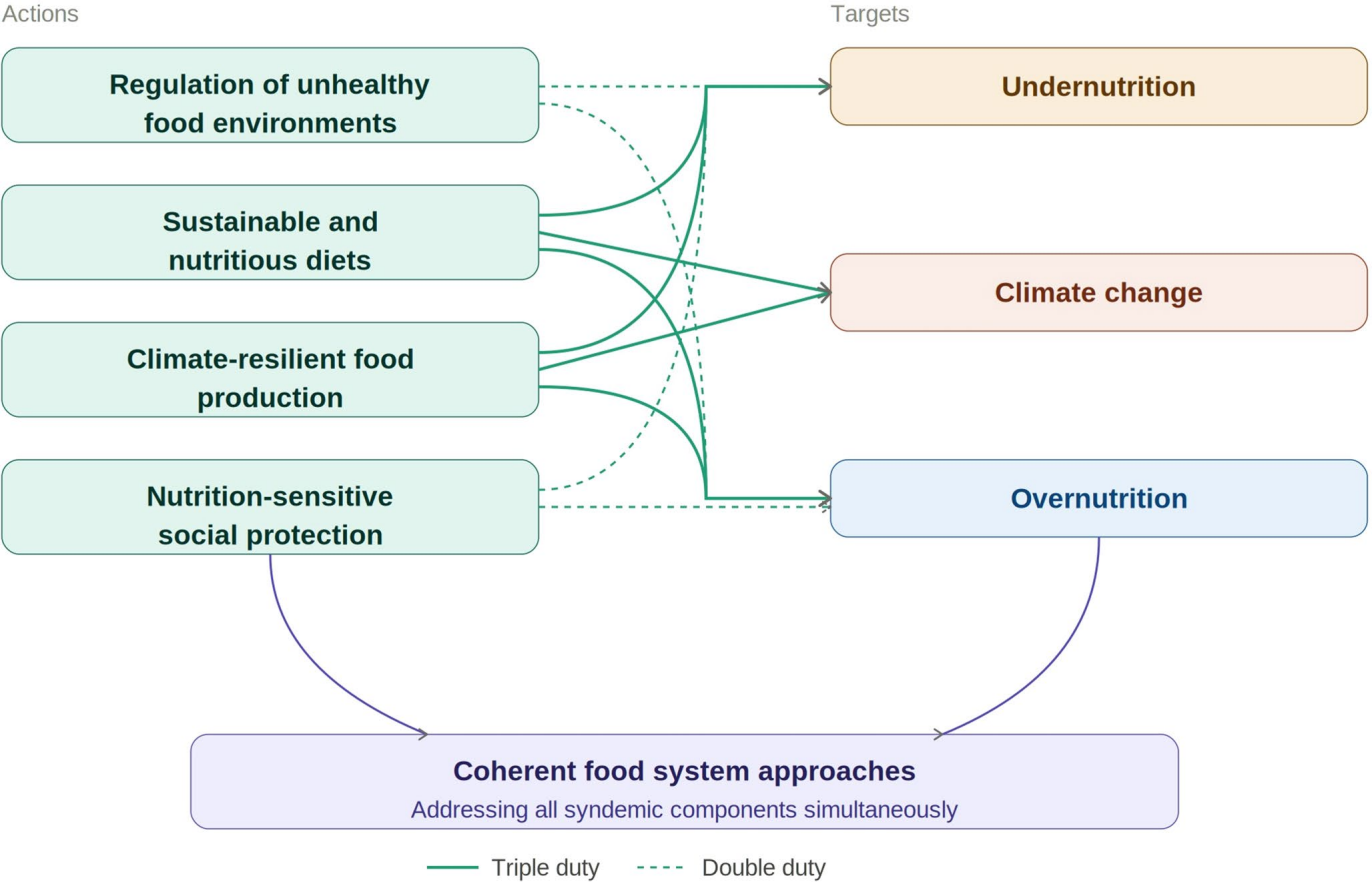
Proposed Double and triple duty action policies frameworks. Double and triple duty actions are policies that simultaneously address two or three components of the global syndemic through coherent food system approaches. Regulation of unhealthy food environments and nutrition-sensitive social protection represent double duty actions, targeting both undernutrition and overnutrition. Promotion of sustainable and nutritious diets and support for climate-resilient, nutrient-dense food production represent triple duty actions, addressing undernutrition, overnutrition, and climate change simultaneously. Solid lines indicate triple duty actions; dotted lines indicate double duty actions

Increasing attention has therefore shifted toward double- and triple-duty actions (Fig. 4), defined as policies that simultaneously address undernutrition, obesity, and climate change through coherent food system approaches. These actions include regulation of unhealthy food environments, promotion of sustainable and nutritious diets, support for climate-resilient and nutrient-dense food production, and expansion of nutrition-sensitive social protection. Although global agencies provide policy frameworks to support such approaches [131], empirical evidence from African settings remains limited [132].

In line with international organisation policy briefs on double- and triple-duty actions to address the global syndemic, several integrated strategies are recommended. First, national dietary guidelines can be leveraged to inform policies and programmes that promote high-quality diets, simultaneously addressing undernutrition, overweight, obesity, NCDs and climate change [130, 131]. However, evidence of their effectiveness remains mixed, with stronger and more consistent impacts reported in high-income settings compared to low- and middle-income countries, highlighting the need for further context-specific research [129, 133]. Secondly, existing policy frameworks often address undernutrition and obesity separately; therefore, integrated national policies targeting nutrition and NCDs are essential to achieve double-duty outcomes and maximize shared benefits [128].

Thirdly, health systems should be strengthened, particularly at the primary care level, to enable early prevention and management of both undernutrition and diet-related NCDs [131], as evidence of interventions targeting children and mothers are already showing promising results in low-income and middle-income countries [128]. Fourthly, humanitarian and emergency nutrition programmes should move beyond a sole focus on food quantity and food security to incorporate diet quality, equity, and long-term health impacts, ensuring that interventions do not inadvertently increase the risk of unhealthy diets [128, 131]. In addition, social policies, including improving female education and access to paid maternity leave, are also critical, as they empower women and enhance dietary quality at both individual and household levels. This is particularly important given the heightened vulnerability of women to undernutrition and micronutrient deficiencies due to biological and socio-cultural factors [128, 131].

Finally, urban planning and food system policies can support active transport systems and improve access to healthy diets through strategies such as urban agriculture a direct farm-to-consumer markets, while reducing reliance on ultra-processed foods [10, 131]. These integrated approaches can generate co-benefits across the double burden of malnutrition and contribute to climate change mitigation by lowering greenhouse gas emissions from food processing and transport sectors [10]. Furthermore, climate-smart agriculture, urban design, and transport policies can enhance the production and accessibility of nutrient-rich foods while reducing emissions, thereby addressing malnutrition, non-communicable diseases, and the adverse effects of climate change in a coordinated manner [10],

## Strengths and Limitations

This narrative review provides an updated synthesis of recent evidence (2019–2025) on the intersecting burdens of undernutrition, overweight and obesity, and climate change in Sub-Saharan Africa. By drawing on a broad range of sources across nutrition, climate, and food systems research, it adopts a systems-oriented perspective that reflects the complexity of the global syndemic. In doing so, it brings together diverse strands of evidence to highlight emerging patterns of convergence between these conditions at population, household, and individual levels, extending insights from earlier work.

The interpretive nature of this review also introduces limitations. As a narrative synthesis, the selection and interpretation of evidence may be subject to bias, and the findings do not provide the quantitative precision of a systematic review or meta-analysis. The included studies are heterogeneous in design, setting, and outcome measures, which limits direct comparability. In addition, evidence remains uneven across regions of Sub-Saharan Africa, and some recent developments may not have been fully captured given the rapidly evolving nature of research in this field. Finally, the complex and interrelated pathways linking climate change, food systems, and nutritional outcomes are not always directly observable, which constrains causal interpretation.

## Conclusion

Over the past five decades, SSA has undergone a profound nutritional and environmental transition. Since the 1970 s, persistent undernutrition has increasingly coexisted with rising overweight and obesity, while climate change has emerged as a major and accelerating driver of food insecurity. Evidence from 2019 to 2025 shows that these challenges now overlap within the same populations, households, and individuals, forming a reinforcing global syndemic of undernutrition, obesity, and climate change.

Despite targeted interventions, undernutrition remains widespread, obesity prevalence continues to increase, and climate-related shocks have intensified in recent years. These intersecting pressures are already contributing to growing burdens of NCDs, sustained vulnerability to infectious diseases, and widening health inequities. Under current trajectories, Africa is unlikely to meet global and regional nutrition and health targets set for 2030.

This review identifies important advances in surveillance, analytical methods, and early warning systems, alongside increasing recognition of integrated policy approaches. However, major gaps persist, including limited longitudinal data, insufficient integration of nutrition and climate information systems, and weak translation of evidence into coordinated action.

Addressing the global syndemic in Africa requires a shift from siloed interventions toward context-specific, multisectoral strategies. Investment in double- and triple-duty actions that simultaneously address undernutrition, obesity, and climate change, while strengthening implementation and evaluation capacity, is urgently needed. Aligning food systems, health systems, and climate adaptation efforts offers a critical opportunity to reduce preventable disease, protect vulnerable populations, and support equitable and sustainable health outcomes across the continent.

## Key References

- Tamir TT, Mekonen EG, Workneh BS, Techane MA, Terefe B, Zegeye AF. Overnutrition and associated factors among women of reproductive age in Sub-Saharan Africa: A hierarchical analysis of 2019–2023 standard demographic and health survey data. Nutrition [Internet]. 2024 Dec 1 [cited 2025 Nov 6];128:112563. Available from: https://www.sciencedirect.com/science/article/pii/S0899900724002120.
  - ◦A secondary analysis of 2019–2023 multi-country Demographic and Health Survey datasets in SSA, highlighting rising burden of overnutrition.
- Gizaw G, Wells JC, Argaw A, Olsen MF, Abdissa A, Asres Y, Challa F, Berhane M, Abera M, Sadler K, Boyd E, Friis H, Girma T, Wibaek R. Associations of early childhood exposure to severe acute malnutrition and recovery with cardiometabolic risk markers in later childhood: 5-year prospective matched cohort study in Ethiopia. The American Journal of Clinical Nutrition [Internet]. 2025 Feb 1 [cited 2026 Feb 1];121(2):343–54. Available from: https://www.sciencedirect.com/science/article/pii/S0002916524014680.
  - ◦A prospective matched cohort study provides longitudinal evidence linking early-life undernutrition with later cardiometabolic risk markers, directly supporting the syndemic concept of shared biological pathways between undernutrition and NCDs.
- Alaba OA, Chiwire P, Siya A, Saliu OA, Nhakaniso K, Nzeribe E, Okova D, Lukwa AT. Socio-Economic Inequalities in the Double Burden of Malnutrition among under-Five Children: Evidence from 10 Selected Sub-Saharan African Countries. International Journal of Environmental Research and Public Health. 2023 Jan;20(8):5489. doi:10.3390/ijerph20085489.
  - ◦Analysis of multi-country Demographics and Health Survey data in SSA, highlighting socioeconomic and inequality driven determinants of the double burden of malnutrition.
- Southern African Development Community (SADC), United Nations Children’s Fund (UNICEF). Climate change and child malnutrition: A double threat to resilience in Southern Africa [Internet]. Gaborone, Botswana: Southern African Development Community (SADC); 2025 [cited 2025 Dec 17]. Report No. Available from: https://www.sadc.int/document/climate-change-and-child-malnutrition-double-threat-resilience-southern-africa.
  - ◦This policy brief explicitly links climate change with child malnutrition in Southern Africa and Its policy-oriented recommendations demonstrate applied, multisectoral strategies that can address the global syndemic.
- Tapkigen J, Harding S, Pulkki J, Atkins S, Koivusalo M. Climate change-induced shifts in the food systems and diet-related non-communicable diseases in sub-Saharan Africa: a scoping review and a conceptual framework. BMJ Open. 2024 Jun 18;14(6):e080241. doi:10.1136/bmjopen-2023-080241 PubMed PMID: 38890143; PubMed Central PMCID: PMC11191816.
  - ◦A scoping review and conceptual framework explicitly connects climate-induced food system shifts to diet-related NCDs in SSA, mapping the structural pathways that unify obesity, undernutrition, and climate change.
- Ngadaya E, Mosses A, Leyna G, Solomon D, Msola H, Said FA, Masanja H, Kagaruki G, Mwiru R, Salmin A, Isangula K, Kiungai M, Kombo KM, Mchau G, Ngegba J, Codjia P. Prevalence and Determinants of Double and Triple Burden of Malnutrition Among School Going Children and Adolescents in Zanzibar, 2022 [Internet]. medRxiv; 2025 [cited 2025 Sep 29]. p. 2025.02.28.25323109. Available from: https://www.medrxiv.org/content/10.1101/2025.02.28.25323109v1 doi:10.1101/2025.02.28.25323109.
  - ◦Reports coexistence of undernutrition, overweight/obesity, and anaemia among children and adolescents.

## Data Availability

All data are from published sources cited in the manuscript.

## References

[1] Nations, Unies, editors. Report on the world social situation, 1993 [Internet]. New York: United Nations; 1993. Available from: https://digitallibrary.un.org/record/172547?v=pdf

[2] The nutritional situation in the african region: Challenges and perspectives [Internet], Brazzaville. Republic of Congo: World Health Organization; 2004 [cited 2025 Dec 17]. Report No.: AFR/RC54/RT/1. Available from: https://iris.who.int/bitstream/handle/10665/93126/AFR.RC54.RT.1.pdf?sequence=1

[3] Popkin BM, Adair LS, Ng SW. Global nutrition transition and the pandemic of obesity in developing countries. Nutr Rev. 2012;70(1):3–21. 10.1111/j.1753-4887.2011.00456.x. 22221213 10.1111/j.1753-4887.2011.00456.x PMC3257829

[4] Steyn NP, Mchiza ZJ. Obesity and the nutrition transition in Sub-Saharan Africa. Ann N Y Acad Sci. 2014;1311(1):88–101. 10.1111/nyas.12433. 24725148 10.1111/nyas.12433

[5] Wojcicki JM. The double burden household in sub-Saharan Africa: maternal overweight and obesity and childhood undernutrition from the year 2000: results from World Health Organization Data (WHO) and Demographic Health Surveys (DHS). BMC Public Health. 2014;14(1):1124. 10.1186/1471-2458-14-1124. 25361562 10.1186/1471-2458-14-1124 PMC4233103

[6] Niang I, Ruppel OC, Abdrabo MA, Essel A, Lennard C, Padgham J, Urquhart P. Africa. In: Barros VR, Dokken DJ, Mach KJ, Mastrandrea MD, Bilir TE, Chatterjee M, Ebi KL, Estrada YO, Genova RC, Girma B, Kissel ES, Levy AN, MacCracken S, Mastrandrea PR, White LL, editors. Climate Change 2014: Impacts, Adaptation, and Vulnerability. Contribution of Working Group II to the Fifth Assessment Report of the Intergovernmental Panel on Climate Change [Internet]. IPCC / Cambridge University Press; 2014 [cited 2025 Dec 17]. pp. 1199–265. Available from: https://www.ipcc.ch/report/ar5/wg2/africa/

[7] Trisos CH, Adelekan IO, Totin E, Ayanlade A, Efitre J, Gemeda A, Kalaba K, Lennard C, Masao C, Mgaya Y, Ngaruiya G, Olago D, Simpson NP, Zakieldeen S. Africa. In: Hans-Otto Pörtner, Melinda M. B. Tignor, Elvira S. Poloczanska, Katja Mintenbeck, Andrés Alegría, Marlies Craig, Stefanie Langsdorf, Sina Löschke, Vincent Möller, Andrew Okem, Bardhyl Rama, editors. Climate Change 2022: Impacts, Adaptation and Vulnerability. Contribution of Working Group II to the Sixth Assessment Report of the Intergovernmental Panel on Climate Change [Internet]. Cambridge University Press; 2022 [cited 2025 Nov 25]. pp. 1285–455. Available from: https://www.ipcc.ch/report/ar6/wg2/chapter/chapter-9/

[8] World Bank Group. The Road to Food Security: How Targeted Transport Investments Can Reduce Waste and Food Prices [Internet]. 2025 [cited 2025 Nov 28]. Available from: https://www.worldbank.org/en/news/feature/2025/05/20/the-road-to-food-security-how-targeted-transport-investments-can-reduce-waste-and-food-prices

[9] Southern African Development Community (SADC), United Nations Children’s Fund (UNICEF). Climate change and child malnutrition: A double threat to resilience in Southern Africa [Internet]. Gaborone, Botswana: Southern African Development Community (SADC); 2025 [cited 2025 Dec 17]. Report No. Available from: https://www.sadc.int/document/climate-change-and-child-malnutrition-double-threat-resilience-southern-africa

[10] Swinburn BA, Kraak VI, Allender S, Atkins VJ, Baker PI, Bogard JR, et al. The global syndemic of obesity, undernutrition, and climate change: The Lancet Commission report. Lancet. 2019;393(10173):791–846. 10.1016/S0140-6736(18)32822-8. 30700377 10.1016/S0140-6736(18)32822-8

[11] Ziraba AK, Fotso JC, Ochako R. Overweight and obesity in urban Africa: A problem of the rich or the poor? BMC Public Health. 2009;9(1):465. 10.1186/1471-2458-9-465. 20003478 10.1186/1471-2458-9-465 PMC2803188

[12] Grace K, Davenport F, Funk C, Lerner AM. Child malnutrition and climate in Sub-Saharan Africa: an analysis of recent trends in Kenya. Appl Geogr. 2012;35(1):405–13. 10.1016/j.apgeog.2012.06.017.

[13] Haggblade S, Me-Nsope NM, Staatz JM. Food security implications of staple food substitution in Sahelian West Africa. Food Policy. 2017;71:27–38. 10.1016/j.foodpol.2017.06.003.

[14] Lobell DB, Schlenker W, Costa-Roberts J. Climate trends and global crop production since 1980. Science. 2011;333(6042):616–20. 10.1126/science.1204531. 21551030 10.1126/science.1204531

[15] World Health Organization. Obesity and overweight [Internet]. 2025 [cited 2025 Nov 23]. Available from: https://www.who.int/news-room/fact-sheets/detail/obesity-and-overweight

[16] Okunogbe A, Nugent R, Spencer G, Powis J, Ralston J, Wilding J. Economic impacts of overweight and obesity: current and future estimates for 161 countries. BMJ Glob Health. 2022. 10.1136/bmjgh-2022-009773. 36130777 10.1136/bmjgh-2022-009773 PMC9494015

[17] Tamir TT, Mekonen EG, Workneh BS, Techane MA, Terefe B, Zegeye AF. Overnutrition and associated factors among women of reproductive age in Sub-Saharan Africa: a hierarchical analysis of 2019–2023 standard demographic and health survey data. Nutrition. 2024;128:112563. 10.1016/j.nut.2024.112563. 39303379 10.1016/j.nut.2024.112563

[18] Adam J, Luoga P, Mtawa A, Nyamhanga T. Prevalence and associated factors of overweight and obesity among adult women in Tanzania from the 2022 Tanzania Demographic and Health Survey. Sci Rep. 2025;15(1):33978. 10.1038/s41598-025-11141-4. 41028109 10.1038/s41598-025-11141-4 PMC12485213

[19] Okyere J, Ayebeng C, Dickson KS. State of multi-morbidity among adults in Cape Verde: findings from the 2020 WHO STEPS non-communicable disease survey. J Public Health (Oxf). 2025;47(3):e344–56. 10.1093/pubmed/fdaf031. 40105441 10.1093/pubmed/fdaf031

[20] Yigezu M, Oumer A, Damtew B, Birhanu D, Workie SG, Hamza A, et al. The dual burden of malnutrition and its associated factors among mother-child pairs at the household level in Ethiopia: an urgent public health issue demanding sector-wide collaboration. PLoS One. 2024;19(11):e0307175. 10.1371/journal.pone.0307175. 39495734 10.1371/journal.pone.0307175 PMC11534222

[21] Tsekpetse P, Salu S, Shiuma J, Nambozo B, Makoko BT, Ahinkorah BO. A multivariate non-linear decomposition analysis of urban-rural disparities in overweight/obesity among men aged 20–49 in Ghana. BMC Public Health. 2025;25(1):1973. 10.1186/s12889-025-23238-6. 40437430 10.1186/s12889-025-23238-6 PMC12117721

[22] Mosha MV, Msuya SE, Kasagama E, Ayieko P, Todd J, Filteau S. Prevalence and correlates of overweight and obesity among primary school children in Kilimanjaro, Tanzania. PLoS One. 2021;16(4):e0249595. 10.1371/journal.pone.0249595. 33886578 10.1371/journal.pone.0249595 PMC8061999

[23] Benayad FZ, El Hilali S, Razine R, Idrissi KS, Abouqal R, Belhaj H, et al. Prevalence and predictive determinants of overweight and obesity in children aged 0–24 months in Morocco: a cross-sectional study. Rocz Panstw Zakl Hig. 2023;74(4):395–406. 10.32394/rpzh.2023.0285. 38117003 10.32394/rpzh.2023.0285

[24] Mank I, De Neve JW, Mauti J, Gyengani GA, Somé PA, Shinde S, et al. Prevalence of obesity and anemia among early adolescents in Junior Secondary Schools: a cross-sectional study in Ouagadougou, Burkina Faso. J Sch Health. 2022;92(11):1081–95. 10.1111/josh.13233. 35989492 10.1111/josh.13233

[25] Walle AD, Kebede SD, Adem JB, Enyew EB, Guadie HA, Bekana T, et al. Spatial variations and predictors of overweight/obesity among under-five children in Ethiopia: a geographically weighted regression analysis of the 2019 Ethiopian Mini Demographic and Health Survey. PLoS One. 2024;19(10):e0312025. 10.1371/journal.pone.0312025. 39401190 10.1371/journal.pone.0312025 PMC11472954

[26] Ngadaya E, Mosses A, Leyna G, Solomon D, Msola H, Said FA, et al. Prevalence and determinants of double and triple burden of malnutrition among school going children and adolescents in Zanzibar, 2022 [Internet]. medRxiv. 2025. 10.1101/2025.02.28.25323109.

[27] Sridhar S, Kang JS, Madzorera I, Zulu E, Makasa J, Cross SB, et al. Undernutrition in older children and adolescents in peri-urban Zambia. Front Public Health. 2023. 10.3389/fpubh.2023.1251768. 37818292 10.3389/fpubh.2023.1251768 PMC10562011

[28] World Obesity Federation. World Obesity Atlas 2022 [Internet]. United Kingdom; 2022 [cited 2025 Nov 23]. Report No. Available from: https://www.worldobesity.org/resources/resource-library/world-obesity-atlas-2022

[29] World Health Organization. Malnutrition [Internet]. 2024 [cited 2025 Dec 8]. Available from: https://www.who.int/news-room/fact-sheets/detail/malnutrition

[30] World Food Programme. What is food security? [Internet]. 2025 [cited 2025 Dec 8]. Available from: https://www.wfp.org/stories/food-security-what-it-means-and-why-it-matters

[31] World Health Organization, United Nations Children’s Fund (UNICEF), WHO/World Bank Group Joint Child Malnutrition Estimates (JME) – 2025 edition [Global report] [Internet]. Geneva: World Health Organization., The World Bank. Levels and trends in child malnutrition: UNICEF/ 2025 Jul [cited 2025 Nov 23]. Report No. Available from: https://data.unicef.org/resources/jme/

[32] Khamis AG, Mwanri AW, Ntwenya JE. Prevalence and factors associated with severity of undernutrition among children aged 6–59 months in Tanzania. Food Science & Nutrition. 2025;13(1):e4648. 10.1002/fsn3.4648. 39803299 10.1002/fsn3.4648 PMC11716998

[33] Seboka BT, Alene TD, Ngusie HS, Hailegebreal S, Yehualashet DE, Gilano G, et al. Spatial variations and determinants of acute malnutrition among under-five children in Ethiopia: evidence from 2019 Ethiopian demographic health survey. Ann Glob Health. 2021;87(1):114. 10.5334/aogh.3500. 34900614 10.5334/aogh.3500 PMC8622002

[34] Asmare AA, Agmas YA. Determinants of coexistence of stunting, wasting, and underweight among children under five years in the Gambia; evidence from 2019/20 Gambian demographic health survey: application of multivariate binary logistic regression model. BMC Public Health. 2022;22(1):1621. 10.1186/s12889-022-14000-3. 36028850 10.1186/s12889-022-14000-3 PMC9414138

[35] Tesfaw LM, Dessie ZG. Multilevel multivariate analysis on the anthropometric indicators of under-five children in Ethiopia: EMDHS 2019. BMC Pediatr. 2022;22(1):162. 10.1186/s12887-022-03172-x. 35354391 10.1186/s12887-022-03172-x PMC8966309

[36] Ssentongo P, Ba DM, Ssentongo AE, Fronterre C, Whalen A, Yang Y, et al. Association of vitamin A deficiency with early childhood stunting in Uganda: a population-based cross-sectional study. PLoS One. 2020;15(5):e0233615. 10.1371/journal.pone.0233615. (**PubMed PMID: 32470055; PubMed Central PMCID: PMC7259702**). 32470055 10.1371/journal.pone.0233615 PMC7259702

[37] Gilano G, Hailegebreal S, Sako S, Haile F, Gilano K, Seboka BT, et al. Understanding child wasting in Ethiopia: cross-sectional analysis of 2019 Ethiopian demographic and health survey data using generalized linear latent and mixed models. JMIR Public Health Surveill. 2023;9:e39744. 10.2196/39744. 36753309 10.2196/39744 PMC9947770

[38] Tamir TT, Tekeba B, Aemro A, Wassie M, Mekonen EG. Prevalence, spatial distribution, and determinants of wasting among children under five in Senegal: spatial and multilevel analyses of the 2023 Senegal Demographic and Health Survey. Front Public Health. 2025;13:1543945. 10.3389/fpubh.2025.1543945. PubMed PMID: 40475194; PubMed Central PMCID: PMC12138375. 40475194 10.3389/fpubh.2025.1543945 PMC12138375

[39] Donkor WES, Mbai J, Sesay F, Ali SI, Woodruff BA, Hussein SM, Mohamud KM, Muse A, Mohamed WS, Mohamoud AM, Mohamud FM, Petry N, Galvin M, Wegmüller R, Rohner F, Katambo Y, Wirth JP. Risk factors of stunting and wasting in Somali pre-school age children: results from the 2019 Somalia micronutrient survey. BMC Public Health. 2022;22(1):264. 10.1186/s12889-021-12439-4. 35139826 10.1186/s12889-021-12439-4 PMC8827289

[40] Tamir TT, Tekeba B, Mekonen EG, Zegeye AF, Gebrehana DA. Spatial heterogeneity and predictors of stunting among under five children in Mozambique: a geographically weighted regression. Front Public Health. 2024;12:1502018. 10.3389/fpubh.2024.1502018. PubMed PMID: 39744381; PubMed Central PMCID: PMC11688635. 39744381 10.3389/fpubh.2024.1502018 PMC11688635

[41] Ntawuyirushintege S, Ahmed A, Bucyibaruta G, Siddig EE, Remera E, Tediosi F, et al. Spatiotemporal trends in stunting prevalence among children aged two years old in Rwanda (2020–2024): a retrospective analysis. Nutrients. 2025;17(17):2808. 10.3390/nu17172808. 40944196 10.3390/nu17172808 PMC12430221

[42] Gilano G, Hailegebreal S, Sako S, Seboka BT. Stunting and associated factors among 6–23 months age children in Ethiopia: application of generalized linear latent and mixed modeling. Ecol Food Nutr. 2022;61(5):3–23. 10.1080/03670244.2022.2109023. 10.1080/03670244.2022.2109023 35934984

[43] Beckmann J, Lang C, du Randt R, Gresse A, Long KZ, Ludyga S, et al. Prevalence of stunting and relationship between stunting and associated risk factors with academic achievement and cognitive function: a cross-sectional study with South African primary school children. Int J Environ Res Public Health. 2021;18(8):4218. 10.3390/ijerph18084218. 33923436 10.3390/ijerph18084218 PMC8072858

[44] Budu E, Armah-Ansah EK, Gyawu NO, Tweneboah R, Sekyi-Dickson K, Oga-Omenka C, Otchi EH. Factors associated with inequalities in malnutrition among children in Ghana using the 2022 GDHS and WHO HEAT framework. BMC Public Health. 2025;25(1):2954. 10.1186/s12889-025-24356-x. 40866872 10.1186/s12889-025-24356-x PMC12382195

[45] Robert BN, Cherono A, Mumo E, Mwandawiro C, Okoyo C, Gichuki PM, et al. Spatial variation and clustering of anaemia prevalence in school-aged children in Western Kenya. PLoS One. 2023;18(11):e0282382. 10.1371/journal.pone.0282382. 38011142 10.1371/journal.pone.0282382 PMC10681207

[46] Asmare AA, Agmas YA. Determinants of coexistence of undernutrition and anemia among under-five children in Rwanda; evidence from 2019/20 demographic health survey: Application of bivariate binary logistic regression model. PLoS ONE. 2024;19(4):e0290111. 10.1371/journal.pone.0290111. 38578819 10.1371/journal.pone.0290111 PMC10997128

[47] Aynalem M, Shiferaw E, Adane T, Gelaw Y, Enawgaw B. Anemia in African malnourished pre-school children: A systematic review and meta-analysis. SAGE Open Med. 2022;10:20503121221088433. doi:10.1177/20503121221088433 PubMed PMID: 35371481; PubMed Central PMCID: PMC8968978. 35371481 10.1177/20503121221088433 PMC8968978

[48] Tadesse SE, Zerga AA, Mekonnen TC, Tadesse AW, Hussien FM, Feleke YW, et al. Burden and determinants of anemia among under-five children in Africa: systematic review and meta-analysis. Anemia. 2022;2022(1):1382940. 10.1155/2022/1382940. 36134386 10.1155/2022/1382940 PMC9482935

[49] Elmighrabi NF, Fleming CAK, Dhami MV, Elmabsout AA, Agho KE. A systematic review and meta-analysis of the prevalence of childhood undernutrition in North Africa. PLoS ONE. 2023;18(4):e0283685. 10.1371/journal.pone.0283685. 37023076 10.1371/journal.pone.0283685 PMC10079122

[50] Quamme SH, Iversen PO. Prevalence of child stunting in Sub-Saharan Africa and its risk factors. Clin Nutr Open Sci. 2022;42:49–61. 10.1016/j.nutos.2022.01.009.

[51] UNICEF Eastern and Southern Africa. Nutrition [Internet]. [cited 2025 Nov 23]. Available from: https://www.unicef.org/esa/nutrition

[52] Ritchie H, Roser M, Micronutrient, Deficiency. Our World in Data [Internet]. 2017 Aug 1 [cited 2025 Nov 23]. Available from: https://ourworldindata.org/micronutrient-deficiency

[53] Nestlé Nutrition Institute Africa. MICRONUTRIENT DEFICIENCIES IN AFRICAN ADULTS [Internet]. 2020 [cited 2025 Nov 23]. Available from: https://nnia.nestlenutrition-institute.org/news/article/2020/10/19/micronutrient-deficiencies-in-african-adults

[54] Adeniyi OV, Masilela C, George JA. Prevalence of vitamin D deficiency and its association with cardiometabolic risk factors among healthcare workers in the Eastern Cape province, South Africa; cross-sectional study. Sci Rep. 2024;14(1):4756. 10.1038/s41598-024-54977-y. 38413628 10.1038/s41598-024-54977-y PMC10899187

[55] Omer S. Africa hunger crisis: Facts, FAQs, and how to help. World Vision [Internet]. 2025 May 5 [cited 2025 Nov 11]. Available from: https://www.worldvision.org/hunger-news-stories/africa-hunger-famine-facts

[56] Gustafson S. Hunger on the rise across Africa: 2025 State of Food Security and Nutrition in the World report [Internet]. 2025 [cited 2025 Nov 11]. Available from: https://ssa.foodsecurityportal.org/node/3686

[57] World Food Programme. The State of Food Security and Nutrition in the World (SOFI) Report [Internet]. 2025 [cited 2025 Nov 23]. Available from: https://www.wfp.org/publications/state-food-security-and-nutrition-world-sofi-report

[58] Militao EMA, Uthman OA, Salvador EM, Vinberg S, Macassa G. Food Insecurity and Associated Factors among Households in Maputo City. Nutrients. 2023;15(10):2372. 10.3390/nu15102372. 37242255 10.3390/nu15102372 PMC10220984

[59] Dlamini SN, Mtintsilana A, Craig A, Mapanga W, Norris SA. Food insecurity and coping strategies associate with higher risk of anxiety and depression among South African households with children. Public Health Nutr. 2024;27(1):e116. 10.1017/S1368980024000879. PubMed PMID: 38576137; PubMed Central PMCID: PMC11036448. 38576137 10.1017/S1368980024000879 PMC11036448

[60] Waqo HW, Mekonnen Woldemedihn G, Asfaw ZG. Predictors and regional prevalence of food insecurity in Ethiopia during COVID-19: a multilevel analysis. BMC Public Health. 2025;25(1):1046. 10.1186/s12889-025-22234-0. 40102751 10.1186/s12889-025-22234-0 PMC11917047

[61] Lamontagne E, Folayan MO, Arije O, Enemo A, Sunday A, Muhammad A, Nyako HY, Abdullah RM, Okiwu H, Undelikwo VA, Ogbozor PA, Amusan O, Alaba OA. The effects of COVID-19 on food insecurity, financial vulnerability and housing insecurity among women and girls living with or at risk of HIV in Nigeria. Afr J AIDS Res. 2022;21(4):297–305. 10.2989/16085906.2022.2113107. PubMed PMID: 36189755. 36189755 10.2989/16085906.2022.2113107

[62] Negesse A, Woyraw W, Temesgen H, Teka Y, Yismaw L, Akalu TY, et al. Spatial exploration of non-resilience to food insecurity, its association with COVID-19 and household coping strategies in East Gojjam districts, Northwest Ethiopia, 2020. Sci Rep. 2022;12(1):15511. 10.1038/s41598-022-19963-2. 36109660 10.1038/s41598-022-19963-2 PMC9476421

[63] Humphries H, Lewis L, Lamontagne E, Choonara S, Dikgale K, Yakusik A, Massawe D, Mkhize N, Mzungu F, Karim QA. Impact of COVID-19 public health responses on income, food security and health services among key and vulnerable women in South Africa. Afr J AIDS Res. 2022;21(4):317–29. 10.2989/16085906.2022.2144392. PubMed PMID: 36538540. 36538540 10.2989/16085906.2022.2144392

[64] Yankey O, Essah M, Amegbor PM. The COVID-19 pandemic and self-reported food insecurity among women in Burkina Faso: evidence from the performance monitoring for action (PMA) COVID-19 survey data. BMC Womens Health. 2025;25(1):42. 10.1186/s12905-025-03565-x. PubMed PMID: 39893400; PubMed Central PMCID: PMC11786433. 39893400 10.1186/s12905-025-03565-x PMC11786433

[65] Beck J, Koebach A, Abreu L, Regassa MD, Hoeffler A, Stojetz W, et al. COVID-19 pandemic and food insecurity fuel the mental health crisis in Africa. Int J Public Health. 2024;68:1606369. 10.3389/ijph.2023.1606369. 38283859 10.3389/ijph.2023.1606369 PMC10811217

[66] Azanaw MM, Anley DT, Anteneh RM, Arage G, Muche AA. Effects of armed conflicts on childhood undernutrition in Africa: a systematic review and meta-analysis. Syst Rev. 2023;12(1):46. 10.1186/s13643-023-02206-4. 36922839 10.1186/s13643-023-02206-4 PMC10015806

[67] Alaba OA, Chiwire P, Siya A, Saliu OA, Nhakaniso K, Nzeribe E, et al. Socio-economic inequalities in the double burden of malnutrition among under-five children: evidence from 10 selected sub-saharan African countries. Int J Environ Res Public Health. 2023;20(8):5489. 10.3390/ijerph20085489. 37107770 10.3390/ijerph20085489 PMC10138555

[68] United Nations. What Is Climate Change? [Internet]. United Nations; [cited 2025 Dec 9]. Available from: https://www.un.org/en/climatechange/what-is-climate-change

[69] World Health Organization. Climate change [Internet]. [cited 2025 Dec 9]. Available from: https://www.who.int/health-topics/climate-change

[70] Dunne D. Analysis: Africa’s extreme weather has killed at least 15,000 people in 2023. Carbon Brief [Internet]. 2023 Oct 25 [cited 2025 Nov 11]. Available from: https://www.carbonbrief.org/analysis-africas-extreme-weather-have-killed-at-least-15000-people-in-2023/

[71] Dunne D. Analysis: Africa’s unreported extreme weather in 2022 and climate change. Carbon Brief [Internet]. 2022 Oct 26 [cited 2025 Oct 7]. Available from: https://www.carbonbrief.org/analysis-africas-unreported-extreme-weather-in-2022-and-climate-change/

[72] African Union. Africa Urban Forum: co-creating solutions to make cities habitable for the growing population | African Union [Internet]. 2024 [cited 2025 Nov 28]. Available from: https://au.int/en/pressreleases/20240904/africa-urban-forum-co-creating-solutions-make-cities-habitable-growing

[73] Nel JH, Steyn NP. The nutrition transition and the double burden of malnutrition in sub-saharan African countries: how do these countries compare with the recommended LANCET COMMISSION global diet? Int J Environ Res Public Health. 2022;19(24):16791. 10.3390/ijerph192416791. 36554669 10.3390/ijerph192416791 PMC9779835

[74] Kumma WP, Loha E. Dietary patterns and their association with cardiovascular risk factors in Ethiopia: A community-based cross-sectional study. Front Nutr. 2023;10. 10.3389/fnut.2023.1074296. 10.3389/fnut.2023.1074296 PMC10076605 37032774

[75] Erzse A, Balusik A, Kruger P, Thsehla E, Swinburn B, Hofman K. Commentary on South Africa’s syndemic of undernutrition, obesity, and climate change. South Afr J Sci. 2023;119(3/4). 10.17159/sajs.2023/14776.

[76] Kamiya Y, Kishida T, Tanou M. Precipitation, temperature, and child undernutrition: evidence from the Mali demographic and health surveys 2012–2013 and 2018. J Health Popul Nutr. 2025;44(1):68. 10.1186/s41043-025-00808-3. 40050927 10.1186/s41043-025-00808-3 PMC11887182

[77] Wells JC, Sawaya AL, Wibaek R, Mwangome M, Poullas MS, Yajnik CS, et al. The double burden of malnutrition: aetiological pathways and consequences for health. Lancet. 2020;395(10217):75–88. 10.1016/S0140-6736(19)32472-9. 31852605 10.1016/S0140-6736(19)32472-9 PMC7613491

[78] Ngwira A. Climate and location as determinants of childhood stunting, wasting, and overweight: an application of semiparametric multivariate probit model. Nutrition. 2020;100010. 10.1016/j.nutx.2020.100010. 10.1016/j.nutx.2020.100010 34301371

[79] Mank I, Belesova K, Bliefernicht J, Traoré I, Wilkinson P, Danquah I, et al. The impact of rainfall variability on diets and undernutrition of young children in rural Burkina Faso. Front Public Health. 2021. 10.3389/fpubh.2021.693281. 34616704 10.3389/fpubh.2021.693281 PMC8489680

[80] Yeboah E, Kuunibe N, Mank I, Parisi D, Bonnet E, Lohmann J, et al. Every drop matters: combining population-based and satellite data to investigate the link between lifetime rainfall exposure and chronic undernutrition in children under five years in rural Burkina Faso. Environ Res Lett. 2022;17(5):054027. 10.1088/1748-9326/ac661c.

[81] Ssentongo P, Ba DM, Fronterre C, Chinchilli VM. Village-level climate and weather variability, mediated by village-level crop yield, is associated with linear growth in children in Uganda. BMJ Glob Health. 2020. 10.1136/bmjgh-2020-002696. 33051281 10.1136/bmjgh-2020-002696 PMC7554468

[82] Global Nutrition Report. Global Nutrition Report [Internet]. [cited 2025 Dec 18]. Ethiopia — Country Nutrition Profile. Available from: https://globalnutritionreport.org/resources/nutrition-profiles/africa/eastern-africa/ethiopia/

[83] Kimutai J, Barnes C, Zachariah M, Philip S, Kew S, Pinto I, Wolski P, Koren G, Vecchi G, Yang W, Li S, Vahlberg M, Heinrich D, Arrighi J, Marghidan CP, Thalheimer L, Singh R, Kane C, Raju E, Otto FE. Human-Induced Climate Change Increased 2021–2022 Drought Severity in Horn of Africa [SSRN Scholarly Paper] [Internet]. Rochester, NY: Social Science Research Network; 2024 [cited 2025 Oct 4]. Available from: https://papers.ssrn.com/abstract=470148610.2139/ssrn.4701486

[84] Brear MR, Erzse A, Clacherty G, Seutlwadi L, Mahomedy S, Maleke K, et al. Unhealthy food outlets and outdoor advertisements in urban South African primary school students’ food environments. Health Promot Int. 2024;39(6):daae138. 10.1093/heapro/daae138. 39569489 10.1093/heapro/daae138 PMC11579604

[85] Ndlovu N, Day C, Aagaard -Hansen J, Sartorius B, Hofman K. Assessment of food environments in obesity reduction: a tool for public health action. S Afr Health Rev. 2018;2018(1):115–23. 10.10520/EJC-144dca8702.

[86] Waqo HW, Woldemedihn GM, Asfaw ZG. Multistate Markov model for household’s food insecurity transitions and their influencing predictors during COVID-19 pandemic in Ethiopia. PLoS One. 2025;20(7):e0326854. 10.1371/journal.pone.0326854. 40608774 10.1371/journal.pone.0326854 PMC12225855

[87] World Food Programme. Overweight and Obesity - in the context of COVID-19 | World Food Programme [Internet]. 2020 [cited 2025 Dec 15]. Available from: https://www.wfp.org/publications/overweight-and-obesity-context-covid-19

[88] Vasseur L, VanVolkenburg H, Vandeplas I, Touré K, Sanfo S, Baldé FL. The effects of pandemics on the vulnerability of food security in West Africa—a scoping review. Sustainability. 2021;13(22):12888. 10.3390/su132212888.

[89] Elnaiem AD, Franke MF, Richterman A, Guillaume Y, Vissieres K, Augustin GC, Ternier R, Ivers LC. Food insecurity and risk of cholera: A cross-sectional study and exploratory analysis of potential mediators. PLoS Negl Trop Dis. 2023;17(2):e0010574. 10.1371/journal.pntd.0010574. 36745661 10.1371/journal.pntd.0010574 PMC9934351

[90] World Health Organization. Noncommunicable diseases [Internet]. [cited 2025 Nov 17]. Available from: https://www.who.int/news-room/fact-sheets/detail/noncommunicable-diseases

[91] World Obesity Federation. World Obesity Day [Internet]. [cited 2025 Nov 17]. Obesity and Non–Communicable Diseases (NCDs). Available from: https://www.worldobesityday.org/obesity-and-ncds

[92] Jobe M, Mactaggart I, Bell S, Kim MJ, Hydara A, Bascaran C, et al. Prevalence of hypertension, diabetes, obesity, multimorbidity, and related risk factors among adult Gambians: a cross-sectional nationwide study. Lancet Glob Health. 2024;12(1):e55-65. 10.1016/S2214-109X(23)00508-9. 38097298 10.1016/S2214-109X(23)00508-9 PMC7618049

[93] Akpa OM, Made F, Ojo A, Ovbiagele B, Adu D, Motala AA, et al. Regional patterns and association between obesity and hypertension in Africa. Hypertension. 2020;75(5):1167–78. 10.1161/HYPERTENSIONAHA.119.14147. 32172619 10.1161/HYPERTENSIONAHA.119.14147 PMC7176339

[94] Temu TM, Macharia P, Mtui J, Mwangi M, Ngungi PW, Wanjalla C, et al. Obesity and risk for hypertension and diabetes among Kenyan adults: results from a national survey. Medicine. 2021;100(40):e27484. 10.1097/MD.0000000000027484. 34622879 10.1097/MD.0000000000027484 PMC8500651

[95] Ejigu BA, Tiruneh FN. The link between overweight/obesity and noncommunicable diseases in Ethiopia: evidences from nationwide WHO STEPS survey 2015. Int J Hypertens. 2023;2023:2199853. 10.1155/2023/2199853. 38023617 10.1155/2023/2199853 PMC10667048

[96] Linden E, Hoevenaar-Blom M, Beune E, Darko SN, Ankrah ST, Meeks KAC, et al. Blood pressure change and hypertension incidence among Ghanaians living in rural Ghana, urban Ghana and The Netherlands: a prospective cohort study. eClinicalMedicine. 2025. 10.1016/j.eclinm.2025.103141. 40115171 10.1016/j.eclinm.2025.103141 PMC11925589

[97] Gona P, Gona C, Ballout S, Mapoma C, Rao S, Mokdad A. Trends in the burden of most common obesity-related cancers in 16 Southern Africa development community countries, 1990–2019. findings from the global burden of disease study. Obesity Science & Practice. 2019;10(1):e715. 10.1002/osp4.715. 10.1002/osp4.715 PMC10804346 38264007

[98] Tapkigen J, Harding S, Pulkki J, Atkins S, Koivusalo M. Climate change-induced shifts in the food systems and diet-related non-communicable diseases in sub-Saharan Africa: a scoping review and a conceptual framework. BMJ Open. 2024;14(6):e080241. 10.1136/bmjopen-2023-080241. PubMed PMID: 38890143; PubMed Central PMCID: PMC11191816. 38890143 10.1136/bmjopen-2023-080241 PMC11191816

[99] Edwards M. The Barker Hypothesis. In: Handbook of Famine, Starvation, and Nutrient Deprivation [Internet]. Springer, Cham; 2017 [cited 2026 Jan 31]. pp. 1–21. Available from: https://link.springer.com/rwe/10.1007/978-3-319-40007-5_71-1 doi:10.1007/978-3-319-40007-5_71-1.

[100] Grey K, Gonzales GB, Abera M, Lelijveld N, Thompson D, Berhane M, et al. Severe malnutrition or famine exposure in childhood and cardiometabolic non-communicable disease later in life: a systematic review. BMJ Glob Health. 2021. 10.1136/bmjgh-2020-003161. 33692144 10.1136/bmjgh-2020-003161 PMC7949429

[101] Ogah OS, Oguntade AS, Chukwuonye II, Onyeonoro UU, Madukwe OO, Asinobi A, et al. Childhood and infant exposure to famine in the Biafran war is associated with hypertension in later life: the Abia NCDS study. J Hum Hypertens. 2023;37(10):936–43. 10.1038/s41371-022-00782-x. 36473942 10.1038/s41371-022-00782-x

[102] Gizaw G, Wells JC, Argaw A, Olsen MF, Abdissa A, Asres Y, Challa F, Berhane M, Abera M, Sadler K, Boyd E, Friis H, Girma T, Wibaek R. Associations of early childhood exposure to severe acute malnutrition and recovery with cardiometabolic risk markers in later childhood: 5-year prospective matched cohort study in Ethiopia. Am J Clin Nutr. 2025;121(2):343–54. 10.1016/j.ajcnut.2024.12.014. 39701423 10.1016/j.ajcnut.2024.12.014

[103] Mwene-Batu P, Wells J, Maheshe G, Hermans MP, Kalumuna E, Ngaboyeka G, Chimanuka C, Owino VO, Macq J, Lukula M, Dramaix M, Donnen P, Bisimwa G. Body composition of adults with a history of severe acute malnutrition during childhood using the deuterium dilution method in eastern DR Congo: the Lwiro Cohort Study. Am J Clin Nutr. 2021;114(6):2052–9. 10.1093/ajcn/nqab293. 34582550 10.1093/ajcn/nqab293 PMC8634579

[104] World Health Organization. Communicable Diseases and Severe Food Shortage. In: Communicable Diseases and Severe Food Shortage: WHO Technical Note [Internet]. World Health Organization; 2010 [cited 2025 Nov 17]. Available from: https://www.ncbi.nlm.nih.gov/books/NBK304206/26158188

[105] Ricci H, Schmid D, Kruger S, Terzoni S, Ricci C. Factors associated with childhood undernutrition in sub-Saharan Africa: a systematic review and meta‐analysis. Matern Child Nutr. 2025;22(1):e70083. 10.1111/mcn.70083. 40886100 10.1111/mcn.70083 PMC12893520

[106] Macheka L, Kembo G, Kairiza T. Gender dimensions of the impact of HIV/AIDS on stunting in children under five years in Zimbabwe. BMC Public Health. 2021;21(1):1461. 10.1186/s12889-021-11410-7. 34315445 10.1186/s12889-021-11410-7 PMC8317274

[107] Uwimana A, Alexiou H, Mutoni J, Robert A, Rujeni N, Cani PD, et al. Associations between undernutrition and malaria infection: a case–control study from Rwanda. Malar J. 2025;24(1):335. 10.1186/s12936-025-05583-4. 41088183 10.1186/s12936-025-05583-4 PMC12522248

[108] Eskenazi B, Rauch S, Elsiwi B, Bornman R, Obida M, Brewer A, et al. Undernutrition and antibody response to measles, tetanus and *Haemophilus Influenzae* type b (Hib) vaccination in pre-school south African children: the VHEMBE birth cohort study. Vaccine. 2025;46:126564. 10.1016/j.vaccine.2024.126564. 39665976 10.1016/j.vaccine.2024.126564 PMC11750586

[109] Ritchie H. Half of all child deaths are linked to malnutrition. Our World in Data [Internet]. 2024 Sep 9 [cited 2025 Nov 30]. Available from: https://ourworldindata.org/half-child-deaths-linked-malnutrition

[110] Wambua J, Ali A, Ukwizabigira JB, Kuodi P. Prevalence and risk factors of under-five mortality due to severe acute malnutrition in Africa: a systematic review and meta-analysis. Syst Rev. 2025;14(1):29. 10.1186/s13643-024-02740-9. 39885605 10.1186/s13643-024-02740-9 PMC11780833

[111] Madewell ZJ, Keita AM, Das PMG, Mehta A, Akelo V, Oluoch OB, et al. Contribution of malnutrition to infant and child deaths in Sub-Saharan Africa and South Asia. BMJ Glob Health. 2024. 10.1136/bmjgh-2024-017262. 39638608 10.1136/bmjgh-2024-017262 PMC11624724

[112] Gooding C, Musa S, Lavin T, Sibeko L, Ndikom CM, Iwuagwu S, et al. Nutritional challenges among African refugee and internally displaced children: a comprehensive scoping review. Children. 2024. 10.3390/children11030318. 38539353 10.3390/children11030318 PMC10969283

[113] Wright CY, Kapwata T, Naidoo N, Asante KP, Arku RE, Cissé G, Simane B, Atuyambe L, Berhane K. Climate Change and Human Health in Africa in Relation to Opportunities to Strengthen Mitigating Potential and Adaptive Capacity: Strategies to Inform an African Brains Trust. Annals Global Health. 2024;90(1). 10.5334/aogh.4260. 10.5334/aogh.4260 PMC10836170 38312714

[114] Mehra D, Rael T, Bloem MW. A review of the intersection between climate change, agriculture, health, and nutrition in Africa: costs and programmatic options. Front Sustain Food Syst. 2024. 10.3389/fsufs.2024.1389730.

[115] World Health Organization. Global targets 2030 [Internet]. [cited 2025 Nov 30]. Available from: https://www.who.int/teams/nutrition-and-food-safety/global-targets-2030

[116] United Nations. Sustainable Development Goals [Internet]. [cited 2025 Nov 30]. Goal 2: Zero Hunger. Available from: https://www.un.org/sustainabledevelopment/hunger/

[117] African Union. Malabo Declaration on Accelerated Agricultural Growth and Transformation for Shared Prosperity and Improved Livelihoods [Internet]. Malabo, Equatorial Guinea: African Union Commission; 2014 [cited 2025 Nov 30]. Report No. Available from: https://www.nepad.org/caadp/publication/malabo-declaration-accelerated-agricultural-growth

[118] Centers for Disease Control and Prevention. Obesity [Internet]. 2025 [cited 2025 Dec 20]. Surveillance Systems for Obesity. Available from: https://www.cdc.gov/obesity/data-and-statistics/surveillance-systems.html

[119] Adong P, Bainomugisha E, Dev S. Evaluating machine learning methods for PM2.5 estimation using satellite AOD, low-cost and reference-grade monitors in Kampala. Int J Environ Sci Technol. 2025;22(15):15747–56. 10.1007/s13762-025-06674-0.

[120] Holloway T, Miller D, Anenberg S, Diao M, Duncan B, Fiore AM, et al. Satellite monitoring for air quality and health. Annu Rev Biomed Data Sci. 2021;4(1):417–47. 10.1146/annurev-biodatasci-110920-093120. 34465183 10.1146/annurev-biodatasci-110920-093120

[121] Health Information Systems Programme. University of Oslo. DHIS2 for Climate & Health. DHIS2 [Internet]. [cited 2025 Nov 18]. Available from: https://dhis2.org/climate/

[122] World Health Organization. STEPwise approach to NCD risk factor surveillance (STEPS) [Internet]. [cited 2026 Feb 1]. Available from: https://www.who.int/teams/noncommunicable-diseases/surveillance/systems-tools/steps

[123] AI4SDGs Think Tank. Nutrition Early Warning System (NEWS) [Internet]. [cited 2025 Nov 18]. Available from: https://ai-for-sdgs.academy/case/188

[124] Panjwani S, Jampani M, Sambou MHA, Amarnath G. Leveraging crop yield forecasts using satellite information for early warning in Senegal. Clim Smart Agric. 2024;1(2):100024. 10.1016/j.csag.2024.100024.

[125] Ndagijimana S, Kabano I, Masabo E, Ntaganda JM. Predicting stunting in Rwanda using artificial neural networks: a demographic health survey 2020 analysis [Internet]. F1000Research; 2024 [cited 2025 Nov 18]. Available from: https://f1000research.com/articles/13-12810.12688/f1000research.141458.1 10.12688/f1000research.141458.2 PMC11840296 39981107

[126] World Health Organization. Global action plan for the prevention and control of noncommunicable diseases, 2013–2020. Geneva, Switzerland: World Health Organization; 2013.

[127] World Health Organization. United Nations Decade of Action on Nutrition (2016–2025): Report by the Director–General [Internet]. Geneva. 2022 [cited 2026 Jan 2]. Report No.: EB152/24. Available from: https://apps.who.int/gb/ebwha/pdf_files/EB152/B152_24-en.pdf

[128] Hawkes C, Ruel MT, Salm L, Sinclair B, Branca F. Double-duty actions: seizing programme and policy opportunities to address malnutrition in all its forms. Lancet. 2020;395(10218):142–55. 10.1016/S0140-6736(19)32506-1. 31852603 10.1016/S0140-6736(19)32506-1

[129] Holdsworth M, Kimenju S, Hallen G, Laar A, Oti SO. Review of policy action for healthy environmentally sustainable food systems in sub-Saharan Africa. Current Opinion in Environmental Sustainability. 2023;65:101376. 10.1016/j.cosust.2023.101376.

[130] Springmann M, Spajic L, Clark MA, Poore J, Herforth A, Webb P, et al. The healthiness and sustainability of national and global food based dietary guidelines: modelling study. BMJ. 2020;370:m2322. 10.1136/bmj.m2322. 32669369 10.1136/bmj.m2322 PMC7362232

[131] World Health Organization. Double-duty actions for nutrition: policy brief [Internet]. Geneva, Switzerland: World Health Organization; 2017 [cited 2025 Dec 19]. Report No. Available from: https://www.who.int/publications/i/item/WHO-NMH-NHD-17.2

[132] Burgaz C, Gorasso V, Achten WMJ, Batis C, Castronuovo L, Diouf A, et al. The effectiveness of food system policies to improve nutrition, nutrition-related inequalities and environmental sustainability: a scoping review. Food Secur. 2023;15(5):1313–44. 10.1007/s12571-023-01385-1.

[133] Venegas Hargous C, Strugnell C, Allender S, Orellana L, Corvalan C, Bell C. Double- and triple-duty actions in childhood for addressing the global syndemic of obesity, undernutrition, and climate change: a scoping review. Obes Rev. 2023;24(4):e13555. 10.1111/obr.13555. 36754361 10.1111/obr.13555

